# Size Effect in Hybrid TiO_2_:Au Nanostars for Photocatalytic Water Remediation Applications

**DOI:** 10.3390/ijms232213741

**Published:** 2022-11-08

**Authors:** Fangyuan Zheng, Pedro M. Martins, Joana M. Queirós, Carlos J. Tavares, José Luis Vilas-Vilela, Senentxu Lanceros-Méndez, Javier Reguera

**Affiliations:** 1 BCMaterials, Basque Center for Materials, Applications and Nanostructures, UPV/EHU Science Park, 48940 Leioa, Spain; 2 Centre of Molecular and Environmental Biology (CBMA), University of Minho, 4710-057 Braga, Portugal; 3 Institute for Research and Innovation on Bio-Sustainability (IB-S), University of Minho, 4710-057 Braga, Portugal; 4 Physics Centre of Minho and Porto Universities (CF-UM-UP), University of Minho, 4710-057 Braga, Portugal; 5 LaPMET—Laboratory of Physics for Materials and Emergent Technologies, University of Minho, 4710-057 Braga, Portugal; 6 Macromolecular Chemistry Research Group (LABQUIMAC), Department of Physical Chemistry, Faculty of Science and Technology, University of the Basque Country (UPV/EHU), 48940 Leioa, Spain; 7 Ikerbasque, Basque Foundation for Science, 48009 Bilbao, Spain

**Keywords:** antibiotic degradation, hybrid TiO_2_:Au nanoparticles, visible photocatalysis, water remediation

## Abstract

TiO_2_:Au-based photocatalysis represents a promising alternative to remove contaminants of emerging concern (CECs) from wastewater under sunlight irradiation. However, spherical Au nanoparticles, generally used to sensitize TiO_2_, still limit the photocatalytic spectral band to the 520 nm region, neglecting a high part of sun radiation. Here, a ligand-free synthesis of TiO_2_:Au nanostars is reported, substantially expanding the light absorption spectral region. TiO_2_:Au nanostars with different Au component sizes and branching were generated and tested in the degradation of the antibiotic ciprofloxacin. Interestingly, nanoparticles with the smallest branching showed the highest photocatalytic degradation, 83% and 89% under UV and visible radiation, together with a threshold in photocatalytic activity in the red region. The applicability of these multicomponent nanoparticles was further explored with their incorporation into a porous matrix based on PVDF-HFP to open the way for a reusable energy cost-effective system in the photodegradation of polluted waters containing CECs.

## 1. Introduction

Photocatalysis has received considerable attention in water remediation applications to degrade contaminants of emerging concern (CECs) [1,2,3] such as pesticides, personal care products, or pharmaceuticals [4,5,6]. The prospect of using sunlight as a light source [1,2] makes them highly relevant in the current world energetic crisis [7,8]. One requirement of photocatalysis for this possibility to happen is that the absorbed photons by the photocatalyst should own higher energy than its bandgap under light radiation so that electron-hole (e^−^-h^+^) pairs are generated. Thus, the photogenerated e^−^ and h^+^ migrate to the surface of photocatalysts to react with H_2_O and O_2_ producing highly reactive species such as hydroxyl radical (•OH) and superoxide radical (•O_2_^−^) which react with the pollutants to eventually degrade them into harmless compounds (e.g., CO_2_ and H_2_O) [1,8].

Among several photocatalysts, titanium dioxide (TiO_2_) is one of the most studied due to its remarkable properties: low cost, high stability, large abundance, biocompatibility, and high photocatalytic efficiency [1,2,5,8,9]. In spite of the aforementioned advantages, its wide bandgap (3.0–3.2 eV), which is only excited under UV radiation or near UV region [8], limits its applicability [1,2,10]. Thus, sunlight radiation cannot be used efficiently since just less than 4% of this radiation corresponds to UV [2]. At the same time, the visible and infrared (IR) radiation of the sunlight spectrum remains unused for this purpose [2]. Another setback of using TiO_2_ is the fast recombination of the e^−^ h^+^ pair, reducing its photocatalytic efficiency [1,8]. Different strategies have been used for extending the photocatalytic efficiency of TiO_2_ under sunlight, such as metal and non-metal doping, metal loading, semiconductor combination, co-catalyst loading, and nanocomposite materials [2,8,10]. It has been shown that the functionalization of the TiO_2_ surface with plasmonic nanoparticles allows efficient photocatalytic activities under visible light because of the Schottky junction development and localized surface plasmon resonance (LSPR) [10,11]. The former reduces the recombination rate of the electron-hole pair, whereas the latter contributes to the strong absorption of visible light and the excitation of active charge carriers (hot electrons) [10,11]. Au has been extensively studied due to its excellent optical properties, low toxicity, and physical and chemical stability [8,10,12]. Moreover, the plasmonic resonance of Au is highly tunable depending on the size and shape of the nanoparticles [10,11,12]. Nevertheless, most work uses spherical Au, limiting its sensitizing use to a relatively narrow plasmonic band around 520 nm [13], with just a few studies [13,14,15,16,17,18,19,20,21,22,23] expanding this response to other wavelengths of the visible spectrum by using gold with different morphologies (mainly combined with TiO_2_ macroscopic substrates) such as nanorods [13,14,15,16,17,18,20,22], nanostar [19,21,23] and trigonal nanoprisms [17], or hexagonal nanoprisms [17]. Nanostars (Au with branched morphology), which are particles with the morphology of multiple highly sharp branches protruding from a central core [23], are ideally suited platforms for the synthesis of nanostructured photocatalysts due to their multiple plasmonic electromagnetic hot-spots and high light absorption cross-section [21,23]. Furthermore, they allow LSPR tunability by changing the size of the Au nanostar, concomitant with a change of the nanostar spikes aspect ratio, which can be used to enhance its light absorption from the visible to NIR region [21,24].

Among several wet chemistry-based synthesis methods for Au nanostar preparation, the seed-mediated-growth process is a common method for the synthesis of monodisperse nanostars [25]. Many of these synthesis methods use surfactants or polymers as shape-directing agents [21,24]. However, their presence can reduce the photocatalytic efficiency by blocking the active sites of the photocatalysts [26]. In this sense, surfactant-free methods, mainly based on the use of Ag as a shape-directing agent [27] to synthesize the Au nanostar are highly suited for catalytic applications with variable plasmon resonance from visible to NIR.

In this work, we developed a multistep approach to produce new hybrid Au-sensitised TiO_2_ nanoparticles that are surfactant-free and where the gold component has a size-tunable nanostar morphology. In this multistep approach, Au spherical nanoparticles were initially generated onto TiO_2_ nanoparticles through a deposition–precipitation method, and then further modified (growth) to induce a change in shape by a surfactant-free nanostars synthesis, generating a branched morphology. By changing the synthesis conditions (seeds to growth Au ratio) different sizes of Au NSs were produced which support broadening the absorption band to the whole visible region and a part of the NIR region.

The different versions of these nanoparticles, with different nanostar sizes, were evaluated and compared for their photocatalytic activity under UV and visible light radiation to degrade the antibiotic, ciprofloxacin (CIP). CIP was chosen as the target contaminant as it is one of the most detected antibiotics in different water matrixes [1,28,29] due to its inefficient removal by conventional wastewater treatment plants and, like all antibiotics, due to their risk to generate antimicrobial resistance bacteria in water reservoirs [1]. We prove that these novel nanoparticles are efficient in the removal of CIP under UV and visible light. Moreover, the photocatalytic assay under different wavelengths from the visible to NIR region was also carried out to understand the effect of the increase of size of Au branched nanoparticles. The results show a threshold in the maximum usable illumination wavelength.

Finally, it is essential to incorporate the nanoparticles into a support material [30,31] for a cost-effective way to degrade the pollutants in water treatment and to avoid the possible secondary pollution coming from nanoparticles [32,33]. Poly (vinylidene fluoride) (PVDF) and its copolymers have been widely used as a polymeric substrate to produce membranes, mainly due to their high chemical, mechanical, thermal, and UV stability, related to the stable C-F bonds of the polymer chain [32,33,34]. Here, the best nanoparticles in terms of performance were selected to be incorporated into a porous polymer matrix of poly(vinylidene fluoride-co-hexafluoropropylene) (PVDF-HFP). The excellent performance of the photocatalytic membranes is proven, opening the way to advanced water remediation strategies on broadband pollutant removal.

## 2. Results and Discussion

### 2.1. Nanoparticle Synthesis and Characterisation

Hybrid nanoparticles of Au and TiO_2_ were synthesised following a multistep approach (Section 3.2). Starting with commercial TiO_2_ nanoparticles, Au nanoparticles (NPs) were grown from those nanoparticles through a deposition–precipitation method. As previously described [8], this step generates the formation of small randomly distributed Au spherical nanoparticles attached to the TiO_2_ nanoparticles (TiO_2_:Au-NSph) (Figure S2a,b). EDX mapping was used to confirm the homogeneous distribution of small Au NPs (red colour) on the surface of TiO_2_ (blue colour) in TiO_2_:Au-NSph (Figure S3). In a second step, the Au component of the hybrid nanoparticles was further grown through a seed-mediated-growth process and using Ag, a branch-inducing agent, to generate Au nanostars attached to the TiO_2_ nanoparticles (TiO_2_:Au-NSs). The added Au grows from the Au component of the TiO_2_:Au-NSph because it is a more energetically favorable process as has been shown in other hybrid nanoparticles [35,36,37]. This branched-induction mechanism has the advantage that it is produced through a surfactant-free method offering a non-coated nanoparticle surface, which is advantageous for the catalytic function of the nanoparticles. Based on this method, three different versions of nanoparticles, Sample A, B, and C, were generated with an increasing quantity of Au and, therefore, with the expanding size of the Au nanostar parts.

**Figure 1:**
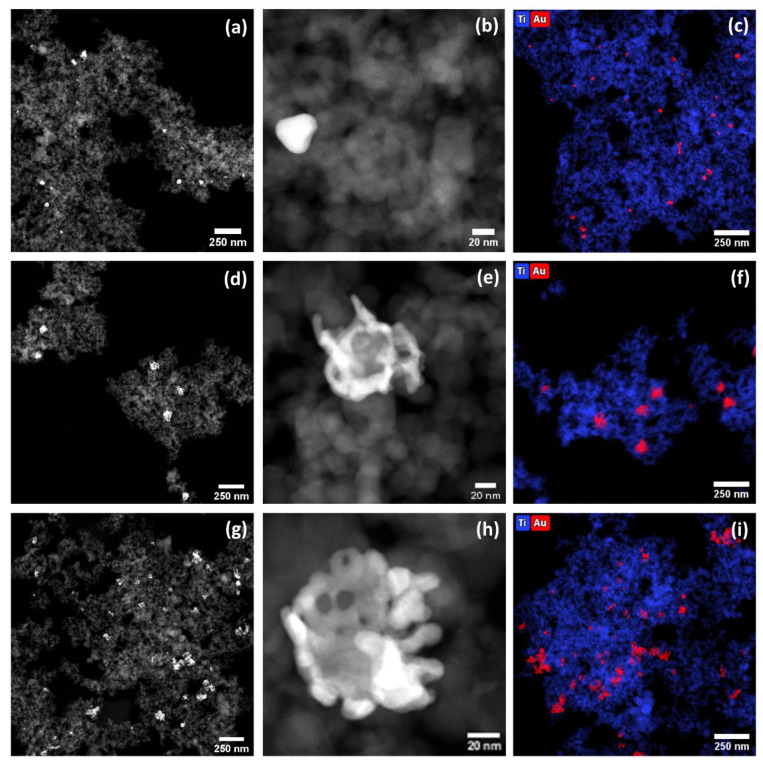
STEM-HAADF micrographs with the different magnifications (**a**,**b**,**d**,**e**,**g**,**h**) and EDX mapping (**c**,**f**,**i**) of TiO_2_:Au-NSs, Sample A (**a**–**c**), Sample B (**d**–**f**), and Sample C (**g**–**i**).

STEM-HAADF was used to assess the morphology of the synthesised nanoparticles. As presented in Figure 1a,b,d,e,g,h, non-spherical Au nanoparticles on the TiO_2_ surface can be detected as high contrast areas in the STEM-HAADF. The shape of the Au particle depends on the synthesis conditions. Increasing the volume ratio of the gold solution to the seed solution generated bigger Au nanostars with more developed tips exhibiting higher aspect ratios (Figure 1b,e,h). Moreover, EDX mapping was used to confirm the presence of Au (red colour) on the surface of TiO_2_ (blue colour) in TiO_2_:Au-NSs-A, B, and –C (Figure 1c,f,i).

**Figure 2:**
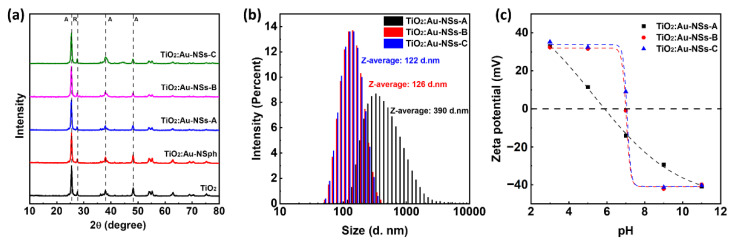
X-ray diffraction spectra of pristine TiO_2_, TiO_2_:Au-NSph, TiO_2_:Au-NSs-A, B, and C nanocomposites and identification of the representative diffraction peaks for anatase (A) and rutile (R) phases (**a**). The intensity size distribution of the TiO_2_:Au-NSs-A, B, and C nanocomposite and respective Z-average hydrodynamic size (**b**). Zeta potential measurements performed at different pHs (3, 5, 7, 9, and 11) for TiO_2_:Au-NSs-A, B, and C nanocomposite (**c**).

Regarding the crystal structure, XRD (Figure 2a) showed the presence of anatase (peaks at 25.3, 37.8, and 48.0°) and rutile (peaks at 27.49°) in all the samples, in good agreement with the literature [8,38]. Moreover, there was no significant difference between the intensities or positions of the peaks from these samples, independent of the Au presence and size. On the other hand, no diffraction peaks of Au were detected in TiO_2_:Au-NSph and TiO_2_:Au-NSs-A, -B and -C, which can be explained by the low amount of Au present in these samples. In addition, XRF was used to detect the amount of Au in the synthesised hybrid nanoparticles (Table S1, Figure S4). The amount of Au with respect to TiO_2_ was 2.38 and 5.83 wt.% in TiO_2_:Au-NSs-B, and –C, respectively, very similar to the theoretical values, considering the quantities of the reagents, indicating a high yield of the Au reduction. Due to the quantification limit of the technique, <1 wt.%, the amount of Au could not be determined for TiO_2_:Au-NSph and TiO_2_:Au-NSs-A although similar results are expected.

The hydrodynamic size for Samples A, B, and C was studied by DLS, (Figure 2b). The results show diameters of 390 ± 13.4, 126 ± 2.9, and 122 ± 0.8 nm for A, B, and C, respectively. These diameters were much smaller than the one of the pristine TiO_2_ nanoparticles previously reported [8] and decreased when increasing the Au-NS size. This is probably due to the different processing that takes place during the growth of Au on the hybrid nanoparticles, with respect to other methods, and the presence of Au nanoparticles over the TiO_2_ surface, preventing the formation of big nanoparticles’ aggregates [8]. Concerning Z-potential, the measurements were performed as a function of the pH and it is presented in Figure 2c. In general, they presented a zero potential around a pH range of 6–7 and a Ζ-potential modulus that rapidly increased when separating from that point to reach values higher than 30 mV for pH below 3 or above 9 where the nanoparticles show superior electrostatic stability [39,40]. When comparing the different nanoparticles, a slight increase of the pH at zero potential was observed when increasing the Au-NS size from Sample A to Samples B and C together with a more pronounced slope, probably due to the smaller aggregate size as indicated in the DLS measurements.

**Figure 3:**
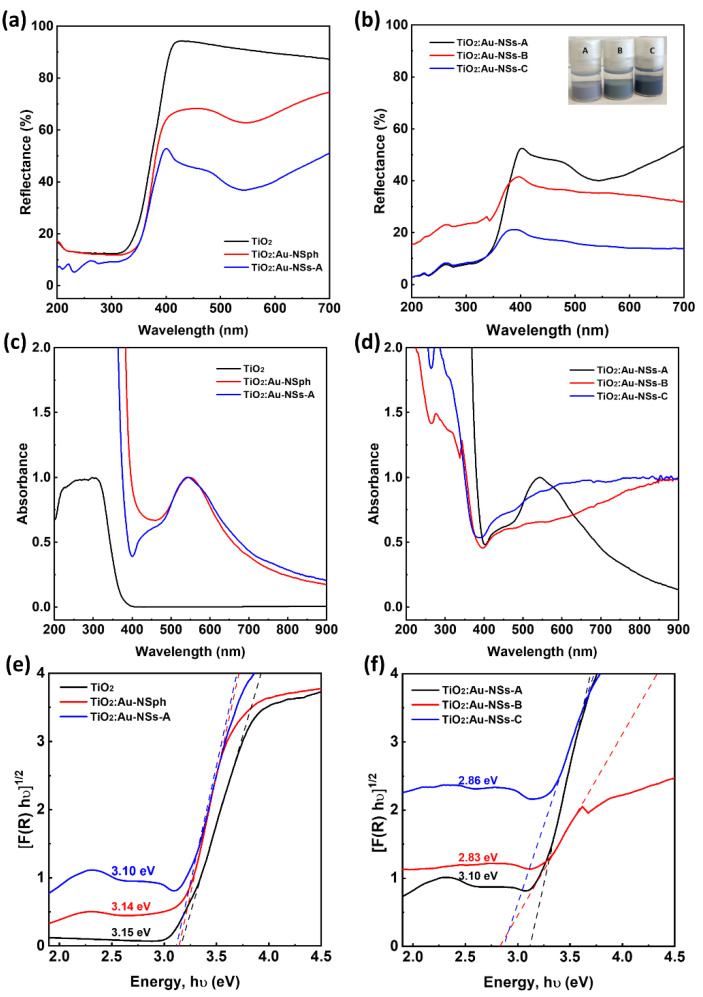
UV-Vis reflectance spectra (**a**,**b**) and UV-Vis absorption spectra (**c**,**d**) of the nanoparticles in the different synthesis steps (**a**,**c**) and nanoparticles with different Au NS sizes (**b**,**d**). Estimation of the bandgap for nanoparticles at different steps of the synthesis (**e**) and nanoparticles with different Au-NS sizes (**f**). (The bandgap is taken as the extrapolation of the linear part at [F(R)hʋ]^1/2^ = 0).

This change in Au morphology affected the optical properties of the nanoparticles. DRS was used to evaluate the optical properties of pure TiO_2_ nanoparticles, TiO_2_:Au-NSph and TiO_2_:Au-NSs (Figure 3a,c,e), and TiO_2_:Au-NSs at different Au-NSs sizes (Figure 3b,d,f)).

When observing the reflectance spectra of the three types of nanoparticles (Figure 3a), all the samples showed similar low reflectance in the UV range (200–400 nm) mainly due to the TiO_2_ high cross-section at the UV. On the other hand, in the visible range (400–700 nm), the pure TiO_2_ nanoparticles had a reflection of ≈87% of the radiation, while the nanohybrids TiO_2_:Au-NSph presented a reflectance below 75% for the same range with a minimum reflectance (≈63%) at 544 nm due to the surface plasmon of Au spherical nanoparticles, which are in line with the literature [8,41]. In the same range, TiO_2_:Au-NSs-A showed a reflectance below 51% due to the higher content of Au and its branched morphology. When comparing the TiO_2_:Au-NSs at different Au-NSs sizes (Figure 3b), reflectance decreased further with the increasing amount and size of gold nanostars. TiO_2_:Au-NSs-B and C, in the same range, presented a reflectance below 32% and 14%, respectively.

The complementary graph of absorbance shows that this one changed significantly when Au was included, with the appearance of a plasmonic peak in the visible region that extended to near IR (Figure 3c). This absorption increased with the concentration of Au and the size of Au branched NPs, generating a broader peak that extended to much higher wavelengths (Samples B and C in Figure 3d). This redshift is in agreement with the literature for homocomponent nanostars [24,27], while the broadening can be due to the interaction with the excess of TiO_2_ nanoparticles affecting its uniformity and morphology.

The bandgap of the samples was estimated from the DRS spectrum by applying Equations (1) and (2) and after line fitting in the linear region 3.3–3.6 eV, as shown in Figure 3e,f. The pure TiO_2_ nanoparticle showed a bandgap of 3.15 eV, typical for TiO_2_ (3.0 to 3.2 eV depending on the ratio of crystalline phases) [42]. The TiO_2_:Au-NSph and TiO_2_:Au-NSs-A showed a lower bandgap than pure TiO_2_: 3.14 and 3.10 eV, respectively. This decrease of the bandgap is related to the absorption of longer wavelengths and has been previously reported for other hybrid systems [8,38,43]. The bandgap reduction was more evident when increasing the Au nanostar size due to their higher absorption in the visible region with values of 2.83 and 2.86 for TiO_2_:Au-NSs-B and C, respectively (Figure 3f).

### 2.2. Photocatalytic Degradation under UV and Visible Radiation

**Figure 4:**
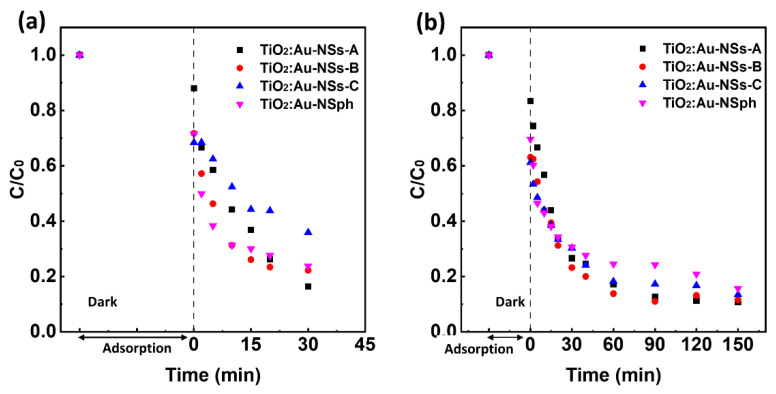
Photocatalytic degradation of CIP (5 mg/L) with TiO_2_:Au-NSph, TiO_2_:Au-NSs-A, B, and C nanoparticles under 30 min of UV radiation (**a**) and 150 min of visible radiation (**b**).

The photocatalytic activity of the synthesized TiO_2_:Au-NSs-A, B, and C nanoparticles was evaluated and compared with TiO_2_:Au-NSph under both UV and visible light radiation in the degradation of CIP under colloidal suspension conditions. Figure 4a,b show the results of photocatalytic experiments under UV and visible radiation, respectively. Table 1 shows the apparent reaction rate constant (*k*) calculated by Equation (3) for the different synthesized nanoparticles. As a control procedure, it should be noted that under the same irradiation conditions of UV or visible light and in the absence of nanoparticles, there was very low photolysis of CIP (Figures S5 and S6).

The photocatalytic assays under UV light had a degradation efficiency of 83, 78, and 64% for A, B, and C nanoparticles, respectively, under the same experimental conditions. The reaction rate constant showed a similar tendency, *k* = 0.053, 0.040, and 0.023 min^−1^ for A, B, and C nanoparticles, respectively. Nanoparticles A presented the best degradation efficiency under UV of the three samples. This result can be rationalized by the lower quantity of TiO_2_ (total nanoparticle mass is kept constant) and the lower active area presented by the photocatalyst when adding the Au to its surface for an increasing quantity of Au. Among these samples, A showed better photocatalytic activity than TiO_2_:Au-NSph, which presented a degradation efficiency of 76%, with *k* = 0.031 min^−1^. The change of the Au spherical morphology to branched morphology reduced the bandgap of TiO_2_ and improved the photocatalytic efficiency.

On the other hand, in the adsorption process in the dark (before irradiation), Samples A, B, and C adsorbed 12%, 28%, and 32% of CIP, respectively. The higher amount of Au on TiO_2_ surface led to a higher CIP adsorption, which agrees with previously reported work [8].

Interestingly, the addition of Au onto the TiO_2_ surface made it possible to produce the photocatalytic degradation of CIP by the nanoparticles under visible light (Figure 4b) due to the improvement of the absorption of longer solar wavelengths as well as the lower bandgap (Figure 3e,f). Under this illumination, all the TiO_2_:Au-NSs nanoparticles presented very similar degradation efficiency, 89%, 88%, and 86%, with *k* = 0.014, 0.013, and 0.009 min^−1^, for A, B, and C, respectively. The nanoparticle TiO_2_:Au-NSph showed slightly lower photocatalytic performance with a degradation efficiency of 84% with *k* = 0.008 min^−1^ compared with A, B, and C. Note here that although TiO_2_:Au-NSs-A, B, and C showed a similar effect under visible radiation, Samples B and C showed a broader plasmonic band that extended deeper into the near IR and could be beneficial for the light-harvesting of higher wavelengths of the sunlight radiation. Finally, the TiO_2_:Au-NSs-A, B, and C after the photocatalytic tests were recovered and assessed by XRD to confirm their stability after photocatalytic application. There was no difference observed in the crystal structure of nanoparticles before (Figure 2a) and after photocatalysis (Figure S7).

Based on the photocatalytic activity results presented, Sample A—with the best photocatalytic performance—was selected as the best candidate to immobilise into a PVDF-HFP-based nanocomposite membrane. Additionally, further experiments were carried out for highly reactive oxygen species (ROS) detection, •OH and ^1^O_2_, in Sample A. Figure S8 shows that the generation of hydroxyl radical (•OH) increased during the 30 min of UV radiation. The result also indicates that the concentration of ^1^O_2_ achieved a maximum at an irradiation time of 15 min, after which the generation of ^1^O_2_ became constant. Therefore, •OH played the dominant role during the photocatalytic degradation of CIP.

On the other hand, a comparison between TiO_2_:Au-NSs-A and previous work using TiO_2_-based plasmonic photocatalysts was performed (Table 2). Due to the different experimental conditions applied in each work, this comparison is not straightforward but allows contextualizing of our results. Although the previous works showed a slightly higher degradation of CIP than our results, they used a much higher amount of plasmonic nanoparticles and intensity of visible radiation (less cost-effective process) or longer degradation time than the one we used, which makes the comparison less straightforward.

### 2.3. Photocatalytic Degradation under Different Wavelengths

To understand the photocatalytic behavior of the nanoparticles after increasing the Au branched morphology, the synthesized TiO_2_:Au-NSs-A, B, and C nanoparticles were evaluated under different wavelengths of light radiation in the visible and NIR region for the degradation of CIP.

**Figure 5:**
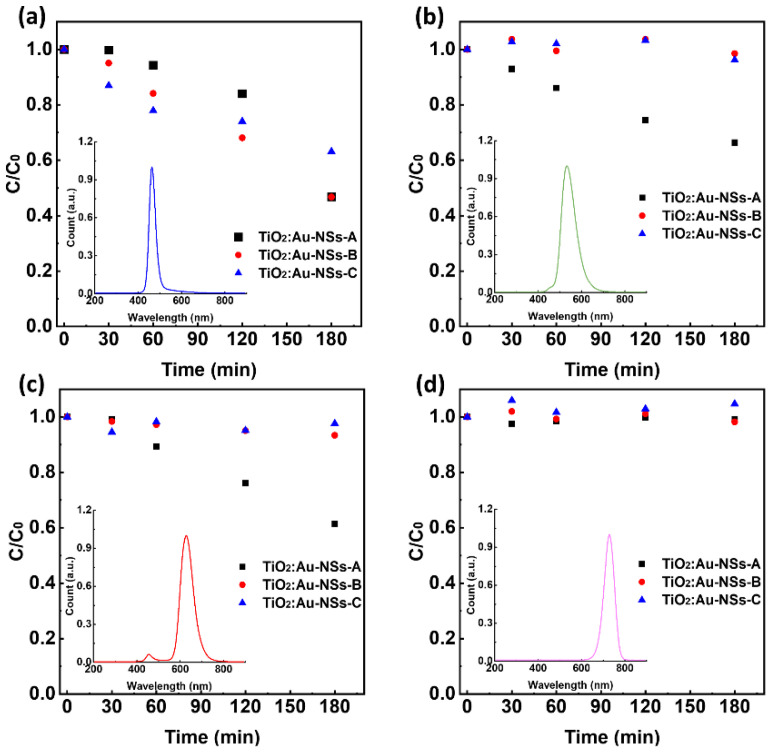
Photocatalytic degradation of CIP (5 mg/L) with TiO_2_:Au-NSs-A, B, and C nanoparticles under 180 min of different wavelengths of light (inset images): blue (**a**), green (**b**), red (**c**), and NIR light (**d**) radiation.

Figure 5a–d show the results of photocatalytic experiments under blue (460 nm), green (530 nm), red (630 nm), and NIR (730 nm) light radiation, respectively. It should be noted that under the same irradiation conditions of these types of light and in the absence of nanoparticles, there was no photolysis of CIP (Figure S9). Table 3 shows the apparent reaction rate calculated by Equation (3).

Regarding the photocatalytic assays under blue light, all nanoparticles showed an excellent degradation activity. Degradation efficiency of 53, 53, and 37% was observed for A, B, and C nanoparticles, respectively, under the same experimental conditions. The rate constant presented a similar tendency, *k* = 0.031, 0.038, and 0.027 min^−1^ for A, B, and C nanoparticles, respectively. This wavelength is lower than the plasmonic band of the Au NSs, however as observed in Figure 3d, the nanoparticles still showed a high absorbance. Interestingly, the nanoparticles showing the highest absorbance are the ones with the lowest degradation activity. This counterintuitive trend can be rationalized by the high absorbance of light by the nanoparticles that block the pass of light deeper in the cuvette and produce the catalytic effect only in the first part of the optical path. In fact, sample C, the one with the highest absorbance, completely blocked the light in a few millimeters. In addition, the highest content of gold, as shown above, produced higher absorption in the dark, and reduced the TiO_2_ surface area (total mass of nanoparticles constant for all experiments) contributing to the lower performance. As the wavelength was increased to green and red light, only TiO_2_:Au-NSs- Sample A was activated in the CIP degradation. A degradation efficiency of 34% and 39% of TiO_2_:Au-NSs-A, with *k* = 0.0024 and 0.0025 min^−1^ for the green and red light radiation was found, respectively. According to the results of photocatalytic assays under NIR light, none of these three nanoparticles could be activated.

For the catalytic process to become activated, electrons from the Au part should gain enough energy from the incident absorbed photon to overpass the Shottky barrier, i.e., the difference between the work function of the Au and the electron affinity in the TiO_2_ as determined by the Schottky–Mott equation (Φ_SB_ = ϕ_M_ − χ_SM_) [47]. Then a lower limit is expected in the absorbed photon energies, i.e., a higher limit in the wavelength. On the other hand, several studies have shown that Φ_SB_ is not only determined by ϕ_M_ and χ_SM_ but also is significantly influenced by interfacial chemistry, giving rise to differences between different types of nanoparticles, in this case with a limit in the 635–735 nm region [48,49].

### 2.4. Membrane Processing and Characterisation

Nonsolvent induces phase separation, NIPS, combined with salt leaching was used to incorporate the synthesized TiO_2_:Au-NSs nanoparticles into the PVDF-HFP polymer matrix and to obtain a porous microstructure. The successful incorporation of the nanoparticles in the polymer matrix extends their reuse, assuring the recovery of the catalyst and, therefore, allowing for a more sustainable and cost-effective application.

**Figure 6:**
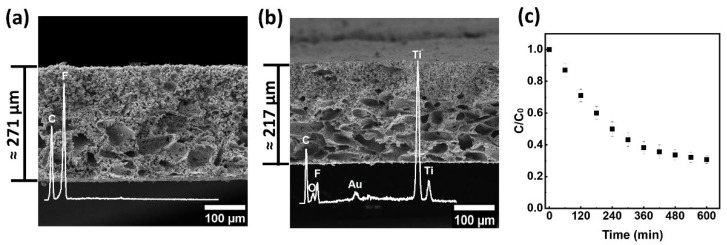
Cross-section SEM images of 0 wt.% (**a**) and 10 wt.% TiO_2_:Au-NSs/PVDF-HFP (**b**). Photocatalytic degradation of CIP (5 mg/L) with 10 wt.% TiO_2_:Au-NSs/PVDF-HFP membrane under 600 min of visible radiation (**c**).

The porous morphology and the presence of nanoparticles were analyzed by SEM (Figure 6a,b). The thickness of the membranes was 271 µm and 217 µm for 0 and 10 wt.% TiO_2_:Au-NSs/PVDF-HFP, respectively. After the incorporation of nanoparticles, the thickness of the membranes was slightly reduced.

High porosity and well-distributed and interconnected pores were observed in both membranes. Notably, the incorporated nanoparticles with 10 wt.% amount did not produce significant changes in the morphology of the pristine membranes. Both membranes presented two ranges of porous distribution due to the polydisperse NaCl grains [50] located mainly in the lower part of the membrane and the additional porous formation by the simultaneous NIPS mechanism [50]. The prepared membrane with 10 wt.% TiO_2_:Au-NSs nanoparticles was tested in the degradation of CIP under visible light (Figure 6c). The membrane did not show a release of nanoparticles to the solution and a CIP degradation efficiency of 69% at 600 min, with *k* = 0.002 min^−1^. Table S2 presents the detected intermediate products of CIP after the photocatalytic assay, confirming the photocatalytic degradation of CIP in the presence of the prepared membrane.

The photocatalytic degradation process was lower when compared to the corresponding colloidal dispersion mainly due to the blocking of part of the nanoparticle surface by the polymer; however, it was enough to obtain a good degradation of the antibiotic and its application facilitates the catalyst recovery and the reusability, and decreases the secondary pollution by nanoparticles to the medium, which makes it an excellent system for environmental application.

## 3. Materials and Methods

### 3.1. Materials

Poly(vinylidene fluoride-co-hexafluoropropylene) (PVDF-HFP, SOLEF^®^ 21216/1001) was purchased from Solvay (Brussels, Belgium). *N*, *N*-dimethylformamide (DMF, ≥99%) and sodium chloride (NaCl, analytical reagent grade) were supplied by Fisher-Scientific (Illkirch, France). Titanium dioxide (TiO_2_) nanoparticles were provided by Evonik Industries AG (Essen, Germany). Hydrogen tetrachloroaurate (III) trihydrate (HAuCl_4_·3H_2_O, 99.99%) was supplied by Alfa Aesar (Stoughton, MA, USA). Sodium hydroxide (NaOH, 98.0–100.5%) was obtained from Panreac (Barcelona, Spain). Hydrochloric Acid (HCl, 37%) was supplied by LABKEM. L-ascorbic acid (AA, ≥99%) and silver nitrate (AgNO_3_, ≥99%) were purchased from Sigma-Aldrich (St. Louis, MO, USA). Milli-Q ultrapure water (resistivity 18.2 MΩ·cm) was used in all experiments. Ciprofloxacin (CIP, ≥98% (HPLG), C_17_H_18_FN_3_O_3_) with maximum light absorption at a wavelength of 277 nm was supplied by Sigma-Aldrich. Absolute ethanol (C_2_H_5_OH, ≥99.5%) were purchased from Sigma-Aldrich.

### 3.2. TiO_2_:Au-NSs Hybrid Nanoparticles Synthesis

The synthesis of TiO_2_:Au-NSs hybrid nanoparticles was divided into two steps. In the first step, Au NPs were grown into TiO_2_ nanoparticles by a deposition–precipitation method (DP) as described by Martins et al. [8] to obtain TiO_2_:Au nanoparticles, where the Au part formed small spherical particles (NSph) of around 5 nm. Briefly, 200 mg of TiO_2_ nanoparticles were dispersed in 40 mL of ultrapure water in a sonication bath for 30 min. Afterwards, a specific volume of HAuCl_4_ (1 mM) was added to achieve an Au loading of 0.05 wt.% and stirred at room temperature for 10 min to disperse the gold precursor homogeneously. Later, NaOH (0.1 M) was added dropwise to obtain a pH = 9 and then stirred for 10 min. Finally, the solution was centrifuged and washed twice with ultrapure water. In the last step, the nanoparticles were dried overnight in an oven at 80 °C and then grounded with a mortar to obtain a fine powder (TiO_2_:Au-NSph).

In a second step, the Au morphology in the TiO_2_:Au-NSph was modified by the seed-mediated-growth process, from spherical Au to star-shaped (NS) Au, using the modified surfactant-free method with the assistance of Ag as a shape-directing agent [51]. Then, 150 mg of TiO_2_:Au-NSph nanoparticles were dispersed in 3.8 mL of ultrapure water in a sonication bath for 30 min as the seed solution. Afterwards, a growth solution was prepared by mixing 18.9 mL of ultrapure water, 19 µL of HCl (1 M), and 95 µL of HAuCl_4_ (50 mM), considering a volume ratio between the gold solution (HAuCl_4_, 50 mM) and seed solution of 0.025. Then, the prepared seed solution was added to this growth solution at room temperature and under moderate stirring. According to the used growth solution volume, 57 µL of AgNO_3_ (10 mM) and 95 µL of AA (100 mM) solution were simultaneously and quickly added to the above mixture under vigorous stirring. The solution rapidly turned from light pink to purplish-grey, indicating the modification of Au morphology from Au sphere to star. This color tended to be more bluish when the ratio of the gold solution to seed solution increased, indicative of the formation of bigger nanostars with higher aspect ratio branches. The obtained samples (TiO_2_:Au-NSs) were centrifuged and washed twice with ultrapure water, resuspended in ultrapure water, and named Sample A for the final application.

In terms of NP size tuning, the final Au star size was controlled by modifying the volume ratio between the gold solution and seed solution in the seed-mediated-growth synthesis step. Samples B and C were obtained by maintaining the volume of the seed solution but modifying the volume of the gold solution (HAuCl_4_, 50 mM), with a volume ratio between gold solution and seed solution of 0.1 and 0.25, for Samples B and C, respectively. The volume of the other reagents in the growth solution, AgNO_3_ and AA solution, were prepared proportionally to the volume of gold solution (HAuCl_4_, 50 mM).

### 3.3. TiO_2_:Au-NSs/PVDF-HFP Membranes Preparation

The most photocatalytic efficient TiO_2_:Au-NSs of the previously synthesized nanoparticles were used to prepare nanoparticle-loaded membranes through a salt leaching technique combined with a non-solvent-induced phase separation (NIPS) following the main guidelines previously described [50] but using different type of coagulation bath. 111 mg of TiO_2_:Au-NSs nanoparticles were dispersed in 9 mL of DMF to obtain a TiO_2_:Au-NSs/PVDF-HFP final mass ratio of 0 and 10 wt.% in an ultrasonication bath for 2 h with control of temperature to achieve a good nanoparticles dispersion. Later, 1 g PVDF-HFP polymer was added to the solution to obtain a PVDF-HFP/DMF concentration of 1:9 *v*/*v*. After dissolving the polymer completely, 5 g of NaCl particles with a diameter of 90 µm were added and stirred for 1 h to achieve a homogeneous distribution of the NaCl particles. Then, the mixed solution was spread onto a glass substrate by a doctor blade with a defined gap of 950 µm. Afterwards, the glass substrate was immersed in an absolute ethanol coagulation bath at room temperature and detached the films. Then, the films were immersed in a distilled water bath at 45 °C to remove possible traces of solvent and dried at room temperature for 24 h. Finally, the film was washed in deionized water for 1 week to remove NaCl particles and then dried at room temperature.

### 3.4. Characterisation Techniques

Transmission electron microscopy (TEM) images were acquired with a JEOL JEM 1400 Plus set up operating at 100 kV in bright field and a Talos (Thermo Scientific, Waltham, MA, USA) system working at 200 kV for the HAADF-STEM and EDX-STEM measurements. To prepare the samples, the nanoparticle powder was dispersed in ultrapure water and sonicated for 1 min, and then 2 μL of the suspension was placed on a 400-mesh carbon-coated copper grid and left to dry at room temperature. The analysis of the images was performed using the ImageJ software package.

To perform diffuse reflectance spectroscopy (DRS), a UV-Visible-NIR Jasco V-770 spectrometer equipped with a 150 mm diameter integrating sphere coated with Spectralon with 1 nm spectral resolution was used. DRS was carried out in the 250–2200 nm wavelength range. A Spectralon reference was used to measure the 100% reflectance, and internal attenuators were used to determine zero reflectance to remove background and noise. The samples saved in ultrapure water were placed in the support and dried at room temperature and the powder samples were placed in a quartz cuvette, sealed, and mounted on a Teflon sample holder before the DRS measurement. The measured reflectance spectra were subsequently converted to Kubelka–Munk (K-M) absorption factors to evaluate the absorption spectra of the samples. This conversion was performed using the K-M Equation (1) [52]:

(1) $$F\left(R\right)={\left(1-R_{\infty }\right)}^{2}/\left(2R_{\infty }\right)$$

where *R_∞_* (*R_Sample_/R_Spectralon_*) corresponds to the reflectance of the sample and *F(R)* is the absorbance.

The sample bandgap was estimated using the Tauc plot Equation (2):

(2) $${\left[F\left(R\right)hʋ\right]}^{1/n}\;versus\;hʋ$$

where *h* is Planck’s constant, *ʋ* the frequency, and *n* the nature of the electronic bandgap transition type, taken as *n* = 2 for indirect transition [53].

Dynamic light scattering (DLS) and Z-potential were measured with a Zetasizer NANO ZS-ZEN3600 (Malvern Instruments Limited, Malvern, UK), equipped with a He–Ne laser (wavelength 633 nm) and backscatter configuration (173°). The nanoparticles were dispersed (1 mg/mL) in ultrapure water in an ultrasonication bath at room temperature for 1 h to avoid aggregation, and each sample was measured five times at pH = 3 to obtain the hydrodynamic diameter. The Z- potential was evaluated at different pH (3, 5, 7, 9, and 11), and each sample was measured five times. HCl (0.1 M) and NaOH (0.1 M) solutions were used to adjust the pH. The resulting particle size was determined using the Smoluchowski model [54]. The manufacturer software (Zetasizer 7.13) was used to estimate the hydrodynamic diameter of the nanoparticles (cumulant diameter), the polydispersity index (PDI), and zeta potential values.

The crystal structure of the nanoparticles was assessed by X-ray diffraction (XRD) using a Philips X’pert PRO automatic diffractometer operating at 40 kV and 40 mA, in theta-theta configuration, secondary monochromator with Cu-Kα radiation (λ = 1.5418 Å) and a PIXcel solid-state detector (active length in 2θ 3.347°). Data were collected from 5 to 80° 2θ, step size 0.026° and time per step of 60 s at room temperature, total measurement time 10 min. Then, 1° fixed soller slit and divergence slit which provided a constant volume of sample illumination, was used.

X-ray fluorescence (XRF) was used to quantify the ratio of Au:TiO_2_ (wt.%). The measurements were obtained by using a MIDEX SD (Spectro, Kleve, Germany) X-ray microfluorescence spectrometer, energy dispersion ED-XRF for elemental analysis. Automatic XYZ tray and collimator changer, X-ray with Mo tube with maximum power 40 W/voltage 48 kV and silicon drift detector (SDD) with 30 mm^2^ area. The calibration and calculations were done by fundamental parameters FP Plus.

A field emission gun scanning electron microscope (FEG-SEM) Hitachi S-4800N operating at 10 kV voltage was used to image the membranes. The samples were coated with a thin layer of gold (≈15 nm) in an Emitech K550X ion-sputter before measurement.

Elemental analysis was carried out by Energy Dispersive X-ray spectroscopy (EDX) using a Carl Zeiss EVO 40 (Oberkochen, Germany) SEM equipped with an EDX Oxford Instrument X-Max detector (Abingdon, UK). The measurement were performed in a high vacuum condition, at a voltage of 20 kV, a current of 100–400 pA and a working distance of 9–10 mm.

Ultrahigh-performance liquid chromatography (UHPLC), coupled with a time-of-flight high-resolution mass spectrometry (TOF-HRMS, Synapt G2 from Waters Cromatografia S.A, Barcelona, Spain) by an electrospray ionisation source in positive mode (ESI+), was used for detecting the products in liquid solution. The chromatographic separation was achieved using an Acquity UPLC BEH C18 column (1.7 μm, 2.1 × 50 mm i.d.) with an Acquity UPLC BEH C18 1.7 μm VanGuard pre-column (2.1 × 5 mm) (Waters Cromatografia S.A.) and a binary solvent A/B gradient (A: water with 0.1% formic acid and B: methanol). The gradient program was as follows: initial conditions were 5% B, raised to 99% B over 2.5 min, held at 99% B until 4 min, decreased to 5% B over the next 0.1 min, and held at 5% B until 5 min for re-equilibration of the system prior to the next injection. A flow rate of 0.25 mL/min was used, with the column temperature at 30 °C, the autosampler temperature at 4 °C, and the injection volume of 5 µL.

### 3.5. Photocatalytic Degradation of Ciprofloxacin under UV and Visible Radiation

The photocatalytic activity of the produced TiO_2_:Au-NSs nanoparticles, A, B, and C, were tested under both UV and visible light radiation. Firstly, the CIP solution of 5 mg/L was prepared and adjusted to pH = 3. Before the degradation assays, 50 mg of nanoparticles as photocatalysts were stirred in 50 mL of CIP solution in the dark for 30 min to achieve an adsorption-desorption equilibrium.

The photocatalytic activity of the produced membranes was tested under visible light radiation using the same CIP solution. Before the degradation assays, the membrane, with a sample area of 18 cm^2^, was immersed and stirred in 50 mL of CIP solution in the dark for 30 min to achieve an adsorption–desorption equilibrium.

The UV degradation of CIP was performed in a photoreactor with eight UV lamps of 8 W, with an emission peak at 365 nm, over 30 min. The suspensions of photocatalysts and CIP were kept stirred in a 100 mL beaker under illumination from the top. The distance between the solution and the lamp was 13.5 cm, and the irradiance at the solution was 3.3 W/m^2^.

For the visible light degradation, a filtered Xenon lamp (sun emulator) with an excitation peak at 550 nm and irradiance of 300 W/m^2^ (spectra in Figure S1) was used, over 150 min for degradation in suspension and 600 min for degradation using membranes. The suspensions of photocatalysts and CIP were stirred in a 100 mL beaker under lateral illumination. The distance between the CIP solution and the lamp was 21 cm.

Aliquots as samples were taken out at different periods during the degradation assays and centrifuged to remove the photocatalysts. Afterwards, the 200 µL of the supernatant in each sample after centrifugation was analyzed by UV-Vis spectroscopy. The absorbance variation of the 277 nm peak of the CIP spectrum was monitored using a microplate reader Infinite 200 Pro in the range of 230 to 450 nm.

The photocatalytic degradation rate was fit to a pseudo-first-order reaction, which is based on the Langmuir–Hinshelwood model described by Equation (3):

(3) $$\ln\left(\frac{C}{C_{0}}\right)=-kt$$

where *C* and *C*_0_ represent the pollutant concentration at time *t* and at the beginning of the photocatalytic assessment respectively, and *k* is the first-order rate constant of the reaction [8].

### 3.6. Photocatalytic Degradation under the Different Wavelengths

The photocatalytic activity of the produced TiO_2_:Au-NSs nanoparticles, A, B, and C was also assessed under different wavelengths of light radiation: blue light (emission peak at 460 nm), green light (emission peak at 530 nm), red light (emission peak at 630 nm), NIR light (emission peak at 730 nm), for ciprofloxacin degradation.

Firstly, the CIP solution of 5 mg/L was prepared and adjusted to pH = 3. After achieving the adsorption–desorption equilibrium of the photocatalysts and CIP solution as described previously (Section 3.5), the degradation of CIP was carried out in a cuvette under different wavelengths of light radiation with an intensity of 0.5 W, over 180 min. The suspensions of photocatalysts and CIP were kept stirred under lateral illumination. The distance between the CIP solution and the lamp was 1 cm.

Aliquots were withdrawn at different times during the degradation assessment, and centrifuged and analyzed using an Agilent (Santa Clara, CA, USA) Cary 60 UV-Vis Spectrophotometer.

## 4. Conclusions

Novel hybrid nanoparticles, TiO_2_:Au-NSs, with an Au branched morphology were synthesized successfully through the surfactant-free method and characterized and tested in photocatalytic assays for ciprofloxacin (CIP) degradation. The characterization results of TEM and DRS show that different sizes of Au NPs with branched morphology were produced by modifying the synthesis conditions, which allowed the tuning of the optical properties of hybrid nanoparticles. When increasing the size of Au NPs with branched morphology, the reflectance of the hybrid nanoparticles decreased from 57% to 13% in the visible region. Additionally, the increase of the size of the Au branched nanoparticles extended the light absorption to the whole visible and part of the NIR region and reduced the bandgap from 3.10 eV to 2.86 eV, respectively.

The photocatalytic assays confirmed that all the synthesized nanoparticles degraded target compound CIP under both UV and white radiation. It was also possible to understand the impact of the size of Au branched nanoparticles in the photocatalytic response. TiO_2_:Au-NSs nanoparticles with smaller Au branched morphology and lowest amount of added Au among these hybrid nanoparticles showed a better photocatalytic performance degrading 83% and 89% ciprofloxacin under UV and visible radiation, respectively.

According to the results, under the different wavelengths of light in the visible and NIR region, the nanoparticles could be activated under blue, green, and red light radiation showing a CIP degradation efficiency of 57%, 34%, and 39%, respectively. However, there was no photocatalytic degradation of CIP under NIR radiation. The bigger size of Au branched nanoparticles limited the light-harvesting of TiO_2_, and reduced the photocatalytic activity, although they showed a broader light absorption in the whole visible and part of the NIR region of sunlight radiation.

The nanoparticles TiO_2_:Au-NSs with lower branching and best performance were selected as the best candidates to incorporate into a PVDF-HFP polymer matrix through the NIPS technique. The membranes were produced successfully and presented high porosity and a well-distributed porous structure.

In short, these results indicate the outstanding performance of the synthesized nanoparticles for water remediation applications in the degradation of persistent contaminants such as ciprofloxacin. Moreover, their successful incorporation into a polymer matrix opens the door to future application in a cost-effective way to degrade a high number of contaminants of emerging concern and other possible applications.

## Figures and Tables

**Table 1:** CIP degradation efficiencies (DE, %) and corresponding apparent reaction rate constants (*k*) under 30 min of UV radiation and 150 min of visible radiation for TiO_2_:Au-NSs-A, B, and C nanoparticles.

Sample	UV		Xenon	
	*k* (min^−1^)	DE (%)	*k* (min^−1^)	DE (%)
TiO_2_:Au-NSs-A	0.053	83	0.014	89
TiO_2_:Au-NSs-B	0.040	78	0.012	88
TiO_2_:Au-NSs-C	0.023	64	0.009	86
TiO_2_:Au-NSph	0.031	76	0.008	84

**Table 2:** Comparison of results between the present work and previous work that used plasmonic nanoparticles to functionalize TiO_2_ for ciprofloxacin (CIP) degradation under visible light.

CIP Concentration (ppm)	Photocatalyst	Cu, Ag or Au Amount (wt.%)	Photocatalyst Concentration (mg/mL)	Irradiation	Efficiency (%)	Time (min)	Ref.
30	Cu/TiO_2_	1.0	0.5	500 W/m^2^	99	180	[44]
80	Cu/TiO_2_	1.0	0.25	500 W/m^2^	85	240	[45]
3.3	Ag/TiO_2_	5.0	0.5	60 W	87	60	[46]
30	Ag/TiO_2_	1.5	0.5	500 W/m^2^	99	240	[44]
30	Au/TiO_2_	1.5	0.5	500 W/m^2^	99	180	[44]
5	Au/TiO_2_	0.5	1.0	98 W/m^2^	45	180	[8]
5	TiO_2_:Au-NSs-A	0.68	1.0	300 W/m^2^	89	150	Present work

**Table 3:** CIP degradation efficiencies (DE, %) and corresponding apparent reaction rate constants (*k*) under 150 min blue, green, red and NIR radiation for TiO_2_:Au-NSs-A, B, and C nanoparticles.

Sample	Blue		Green		Red		NIR	
	*k* (min^−1^)	DE (%)	*k* (min^−1^)	DE (%)	*k* (min^−1^)	DE (%)	*k* (min^−1^)	DE (%)
TiO_2_:Au-NSs-A	0.0031	53	0.0024	34	0.0025	39	-	-
TiO_2_:Au-NSs-B	0.0038	53	-	-	-	-	-	-
TiO_2_:Au-NSs-C	0.0027	37	-	-	-	-	-	-

## Data Availability

Not applicable.

## Supplementary Materials

The following supporting information can be downloaded at: https://www.mdpi.com/article/10.3390/ijms232213741/s1.
