# Supplementary material for: Size Effect in Hybrid TiO2:Au Nanostars for Photocatalytic Water Remediation Applications
Source: Int J Mol Sci. 2022 Nov 8;23(22):13741. doi: 10.3390/ijms232213741 (PMC9692482; doi:10.3390/ijms232213741)
Supplement: Supplementary file 1 [file ijms-23-13741-s001.zip › ijms-2005068-supplementary.pdf]

Supporting Information

Size Effect in Hybrid TiO_2:Au Nanostars for Photocatalytic Water Remediation Applications

Fangyuan Zheng [1], Pedro M. Martins [2,3], Joana M. Queirós [2,3,4], Carlos J. Tavares [4,5], José Luis Vilas-Vilela [1,6], Senentxu Lanceros-Méndez [1,7] and Javier Reguera [1,*]

BCMaterials, Basque Center for Materials, Applications and Nanostructures, UPV/EHU Science Park, 48940 Leioa, Spain
[2] Centre of Molecular and Environmental Biology (CBMA), University of Minho, 4710-057 Braga, Portugal
[3] Institute for Research and Innovation on Bio-Sustainability (IB-S), University of Minho, 4710-057 Braga, Portugal
[4] Physics Centre of Minho and Porto Universities (CF-UM-UP), University of Minho, 4710-057 Braga, Portugal
[5] LaPMET—Laboratory of Physics for Materials and Emergent Technologies, University of Minho, 4710-057 Braga, Portugal
[6] Macromolecular Chemistry Research Group (LABQUIMAC), Department of Physical Chemistry, Faculty of Science and Technology, University of the Basque Country (UPV/EHU), 48940 Leioa, Spain
[7] Ikerbasque, Basque Foundation for Science, 48009 Bilbao, Spain
* Correspondence: javier.reguera@bcmaterials.net

S-1: Xenon Lamp and sunlight spectra

S-2: Morphology of Au in TiO_2:Au-NSph

S-3: EDX mapping of Au in TiO_2:Au-NSph

S-4: Amount of Au wt. % in TiO_2:Au-NSs

S-5: Photolysis of ciprofloxacin under UV radiation

S-6: Photolysis of ciprofloxacin under visible radiation

S-7: X-ray diffraction spectra of TiO_2:Au-NSs-A, B and C nanocomposites after photocatalytic application

S-8: Photogenerated ROS (•OH and $^1O_2$) measurements

S-9: Photolysis of ciprofloxacin under blue, green, red, and NIR light radiation

S-10: Detected degradation products of CIP by HPLC

S-1: Xenon Lamp and sunlight spectra

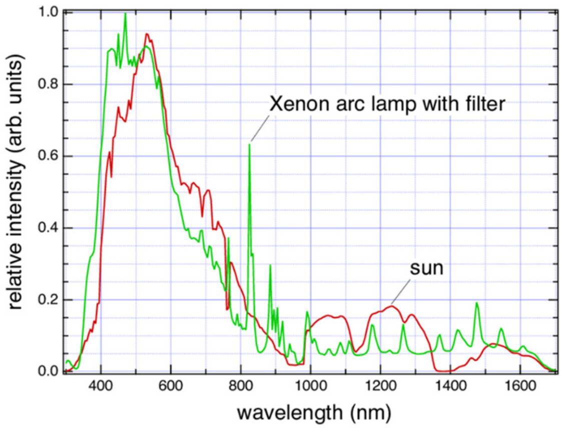

Figure S1. Xenon Lamp arc (with UV filter) and sunlight spectra.

S-2: Morphology of Au in TiO_2:Au-NSph

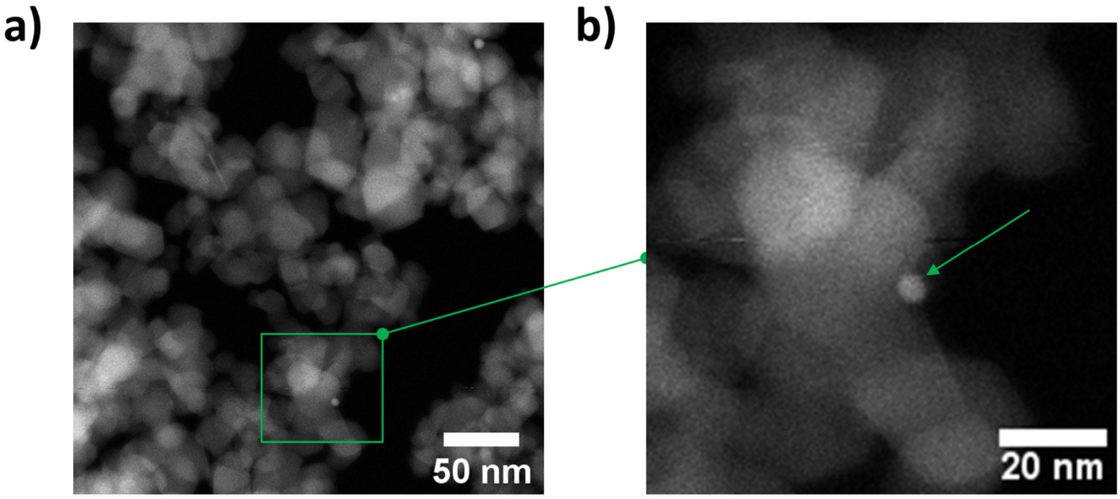

Figure S2. STEM-HAADF micrographs of TiO_2:Au-NSph with different magnifications (**a**,**b**). Au nanoparticles appear as whiter, smaller, and quite circular shapes, scattered through the sample. The zoom-in, with the green arrow, shows one of these Au nanoparticles bound to a TiO_2 nanoparticle surface.

S-3: EDX mapping of Au in TiO_2:Au-NSph

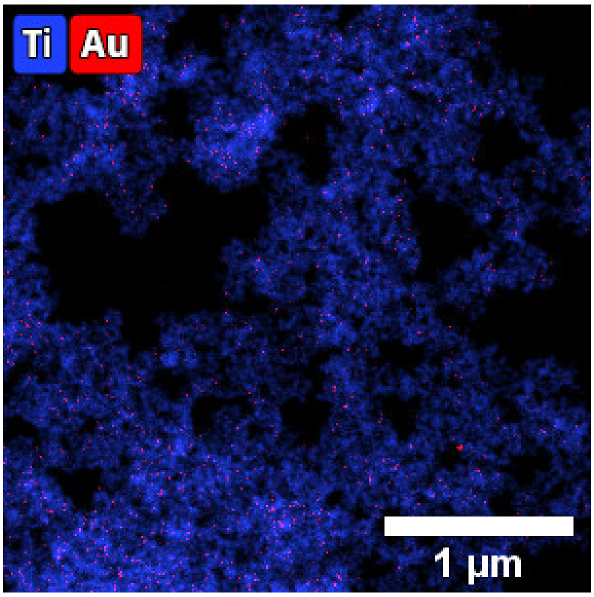

Figure S3. EDX mapping of TiO_2:Au-NSph: Au (red) and Ti (blue).

S-4: Amount of Au wt. % in TiO_2:Au-NSs

Table S1. Amount of Au with respect to TiO_2 (wt. %) in the samples, theoretical value (considering the added reagents in the synthesis reaction) vs. XRF measurement.

Sample	Au:TiO_2 wt. % Theoretical	Au:TiO_2 wt. % XRF
TiO_2:Au-NSph	0.05	*
TiO_2:Au-NSs-A	0.68	*
TiO_2:Au-NSs-B	2.55	2.38
TiO_2:Au-NSs-C	6.30	5.83

* No detected Au since the amount of Au is less than 1% w.t. in sample.

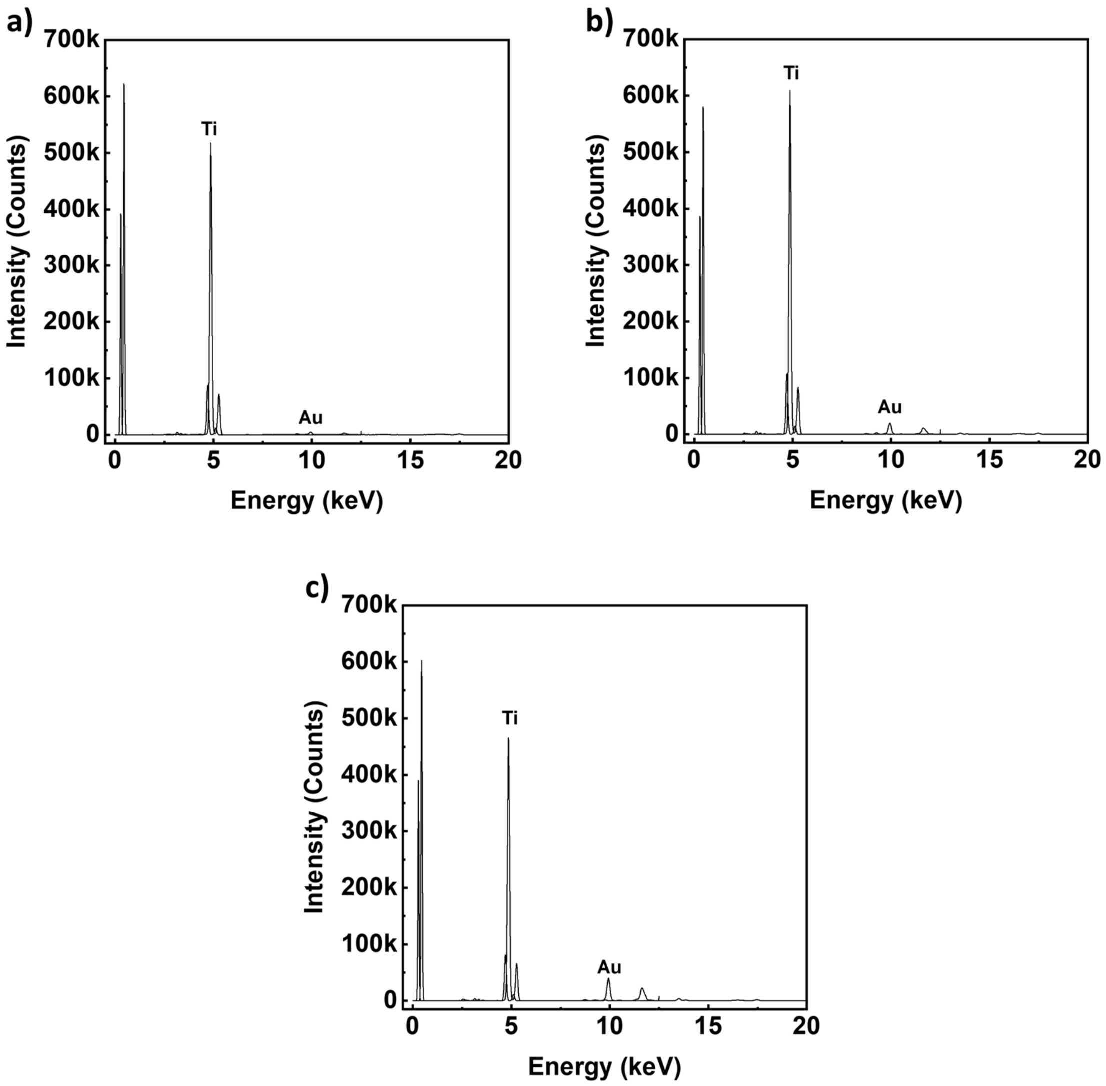

Figure S4. XRF spectrum of TiO_2:Au-NSs-A (**a**), TiO_2:Au-NSs-B (**b**), and TiO_2:Au-NSs-C (**c**).

S-5: Photolysis of ciprofloxacin under UV radiation

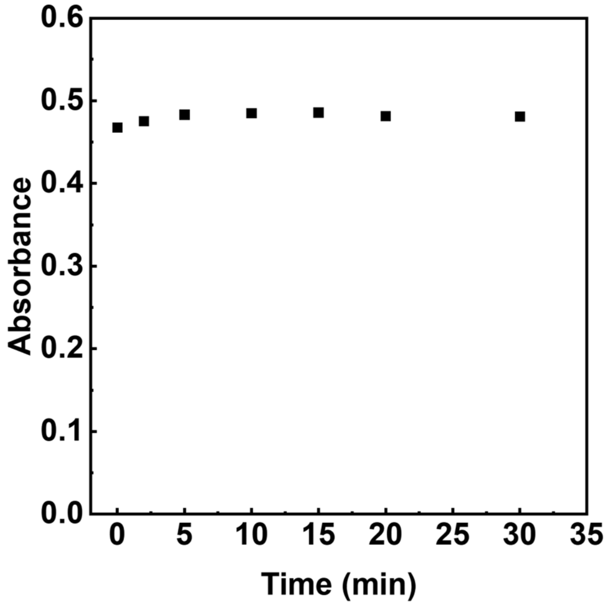

Figure S5. Photolysis assay of CIP (5 mg/L) under 30 minutes of UV radiation.

S-6: Photolysis of ciprofloxacin under visible radiation

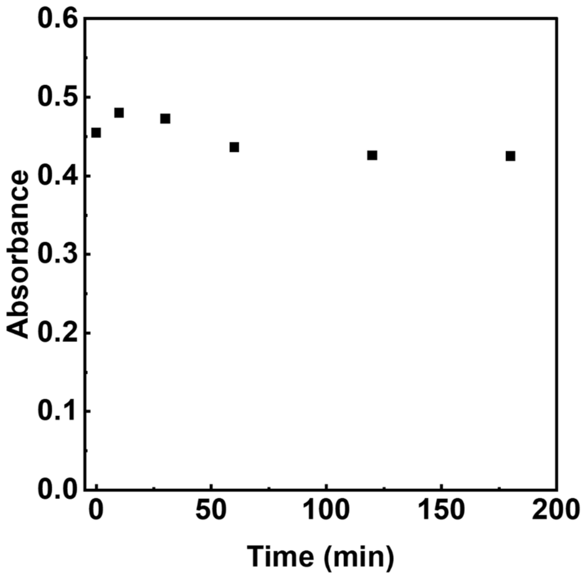

Figure S6. Photolysis assay of CIP (5 mg/L) under 180 minutes of visible radiation.

S-7: X-ray diffraction spectra of TiO_2:Au-NSs-A, B, and C nanocomposites after photocatalytic application

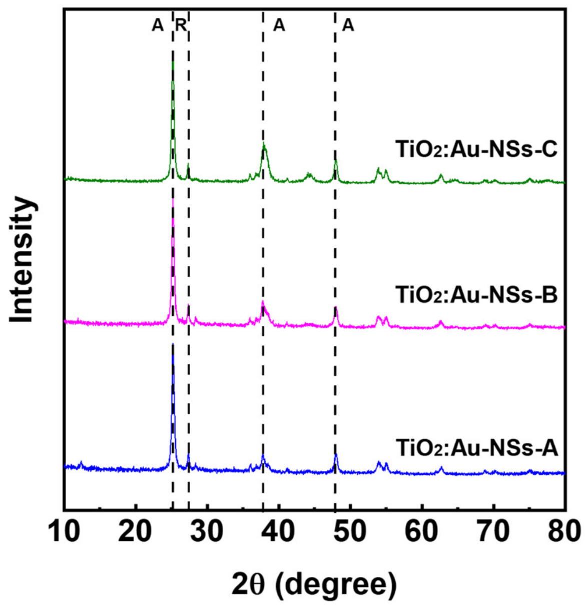

Figure S7. X-ray diffraction spectra of TiO_2:Au-NSs-A, B, and C nanocomposites after photocatalytic application.

S-8: Photogenerated ROS (•OH and $^1O_2$) measurements

- **Detection of hydroxyl radicals (•OH)**

The hydroxyl radicals (•OH) produced after illumination were detected and quantified as previously reported [1] by fluorescence spectroscopy through the hydroxylation reaction of terephthalic acid (TA) to 2-hydroxyterephthalic acid (2-HTA) in the heterogeneous phase.

Firstly, a TA solution (0.5 mM) was prepared by dissolving TA in a dilute NaOH solution (2 mM). Then, 50 mg nanoparticles as photocatalysts were dispersed in 50 mL of TA solution (0.5 mM) and stirred in the dark for 30 min. Afterwards, the suspension was stirred and irradiated for 30 minutes under UV illumination. An aliquot was taken out at different irradiation times and centrifuged to eliminate the nanoparticles. 200 µl of supernatant was taken out and analysed using a microplate reader Infinite 200 Pro. This analysis was carried out by evaluating the emission peak at 425 nm of the 2-HTA which has an excitation wavelength at 315 nm [2] in the fluorescence spectrum. A calibration curve was constructed to quantify the relationship between the fluorescence signal and the generated hydroxyl radicals, using the 2-HTA as a standard.

- **Detection of singlet oxygen ($^1O_2$)**

Singlet oxygen ($^1O_2$) was determined as previously reported [1], as an indirect way to quantify the generated $•O_2^-$. 50 mg of nanoparticles were added to a mixed solution of 40 ml L-histidine solution (0.2 mM) and 10 ml solution of N,N-pnitrosodimethylaniline solution (0.2 mM). Afterwards, the suspension was stirred in the dark for 30 min and then irradiated for 30 min under UV illumination. At different irradiation times, aliquots were taken out, centrifuged, and then analysed using a microplate reader Infinite 200 Pro. This analysis was

performed by evaluating the characteristic band of N,N-p-nitrosodimethylaniline at 440 nm in the UV-Vis spectrum.

The produced $^1O_2$ was quantified from the calibration curve between absorbance and N,N-pnitrosodimethylaniline solution concentration.

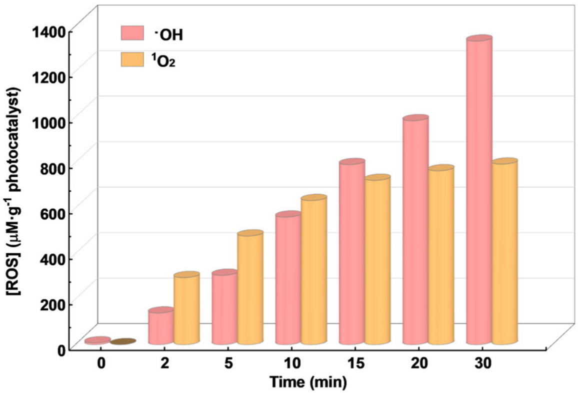

Figure S8. Quantification of photogenerated hydroxyl radical ($^•OH$) and singlet oxygen ($^1O_2$) by TiO_2:Au-NSs-A under 30 minutes of UV radiation.

S-9: Photolysis of ciprofloxacin under blue, green, red, and NIR light radiation

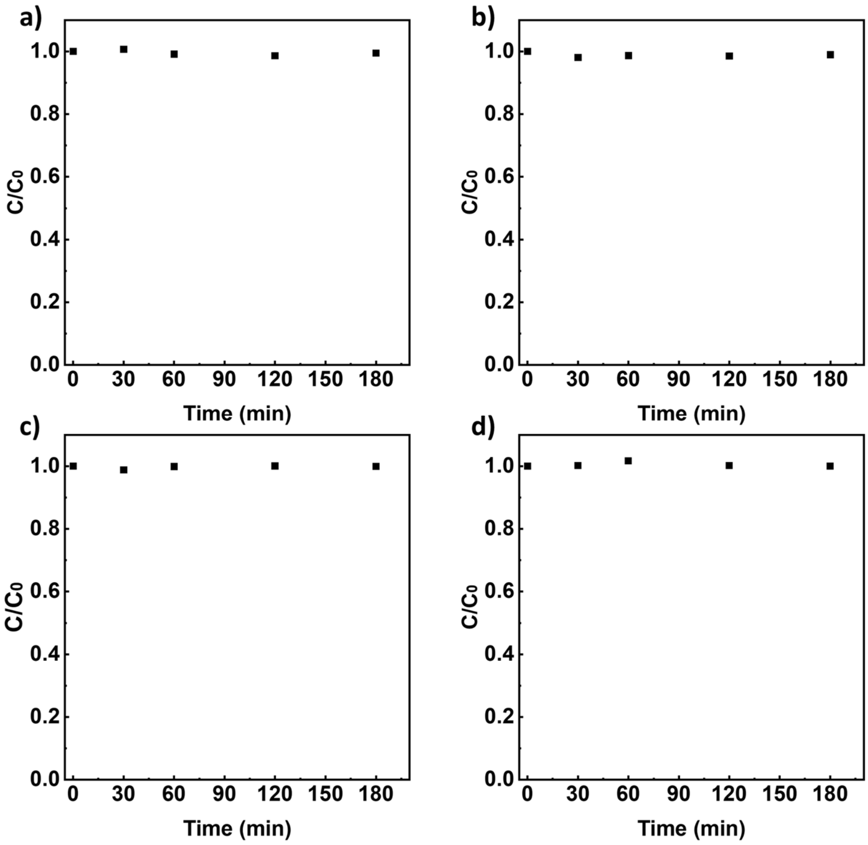

Figure S9. Photolysis assay of CIP (5 mg/L) under 180 minutes of blue (**a**), green (**b**), red (**c**) and NIR light (**d**) radiation.

S-10: Detected degradation products of CIP by HPLC

Table S2. Detected degradation products of CIP.

Detected Molecule	*Detected m/z	Theoretical m/z	Mass Error (mDa)	Mass Error (ppm)
$C_{17}H_{19}N_3O_3F$	332.1412	332.141	0.2	0.5
$C_{17}H_{20}N_3O_4$	330.1451	330.1454	-0.3	-0.9
$C_{16}H_{17}N_3O_4F$	334.1202	334.1203	-0.1	-0.3
$C_{15}H_{17}N_3O_3F$	306.1253	306.1254	-0.1	-0.3
$C_{17}H_{17}N_3O_3F$	330.1257	330.1254	0.3	0.9
$C_{13}H_{12}N_2O_3F$	263.0831	263.0832	-0.1	-0.4
$C_{17}H_{18}N_3O_5$	344.1241	344.1246	-0.5	-1.6
$C_{17}H_{19}N_3O_4F$	348.135	348.136	-1	-2.8
$C_{15}H_{18}N_3O_4$	304.1293	304.1297	-0.4	-1.4
$C_{17}H_{17}N_3O_5F$	362.1146	362.1152	-0.6	-1.7
$C_{17}H_{19}N_3O_5F$	364.1308	364.1309	-0.1	-0.2
$C_{17}H_{17}N_3O_4F$	346.1194	346.1203	-0.9	-2.6

*The masses correspond to protonated molecule [M+H]+.
